# Suitability of the National Health Care Surveys to Examine Behavioral Health Services Associated with Polycystic Ovary Syndrome

**DOI:** 10.1007/s11414-016-9543-6

**Published:** 2016-12-13

**Authors:** Ninive Sanchez

**Affiliations:** School of Social Work, University of Missouri, 712 Clark Hall, Columbia, MO 65211 USA

## Abstract

The National Institutes of Health, Office of Disease Prevention, has described polycystic ovary syndrome (PCOS) as a major public health problem for women in the USA. This study examines the suitability of the National Health Care Surveys, collected by the Centers for Disease Control and Prevention, to understand patient demographics and behavioral health services associated with PCOS-related medical visits. Data were from the 2005–2010 National Ambulatory Medical Care Survey and National Hospital Ambulatory Medical Care Survey. PCOS-related medical visits were identified using the International Classification of Diseases, 9th Revision, Clinical Modification code 256.4. Items on mental health and health education ordered or provided did not meet the National Center for Health Statistics criteria necessary to produce reliable national estimates (i.e., at least 30 unweighted records and a relative standard error <30%). Findings underscore the need to strengthen national surveillance to further understand behavioral health care for patients with PCOS.

## Introduction

While the USA has made considerable advances in women’s health, such as reductions in mortality from chronic diseases like breast cancer and cardiovascular disease, less progress has been made in the detection and treatment of polycystic ovary syndrome (PCOS).^1^ The National Institutes of Health, Office of Disease Prevention, has described PCOS as a major public health problem for women in the USA.^2^ PCOS is a complex, chronic condition characterized by a range of symptoms including irregular or no menstrual periods, excess hair growth on the face and body (hirsutism), weight gain, acne, ovarian cysts, and alopecia.^2^ PCOS can increase women’s risk of type 2 diabetes,^3^ cardiovascular disease,^4^ infertility,^5^ anxiety and depression,^6^^–^ 9, and poor health-related quality of life.^10^ PCOS can begin in adolescence^11^ and worsen across the life course if untreated or poorly managed.^12^^,^ 13

The prevalence of PCOS among adult women (18 to 45 years of age) in the USA is about 7%.^14^ However, research with a community sample of women (27 to 34 years of age) in the UK suggests that the prevalence of PCOS may be even higher, from 7.8 to 20.6%, depending on which of the three diagnostic criteria for PCOS is used. In addition, using two of these criteria, NIH and Rotterdam, about 70% of women with PCOS symptoms are undiagnosed.^15^ Issues contributing to the lack of diagnosis include differences in diagnosis,^15^ particularly among health care providers from different specialties,^16^ and misperceptions among patients and providers that cysts in the ovaries are a key feature of PCOS due to the condition’s misleading name.^17^ The name suggests that the condition is a problem of the ovaries; however, the presence of polycystic ovaries alone does not indicate that a woman has PCOS.^2^ (See the 2012 NIH Evidence-based Methodology Workshop on PCOS for an overview of diagnostic criteria and strengths and limitations of each criteria).^2^ As a result, the 2012 NIH Evidence-based Methodology Workshop on PCOS recommended establishing “multidisciplinary programs to improve public and health care provider awareness and management for women who currently have the syndrome”. 2 ^(p. 8)^

Although large-scale, epidemiological studies to understand the prevalence, phenotypes, and morbidities associated with PCOS are currently unavailable,^2^ national health care data can provide insight into patient demographics and health care associated with PCOS-related medical visits. While researchers have questioned whether young adult women with PCOS are “slipping through the health care cracks,”^18^ ^(p. 1)^ only one study to date has examined health care associated with PCOS-related medical visits using nationally representative health care data.^19^ In that study, Jason used the 2003–2008 data from the National Ambulatory Medical Care Survey (NAMCS) and National Hospital Ambulatory Medical Care Survey (NHAMCS), collected by the National Center for Health Statistics, Centers for Disease Control and Prevention, and found the mean yearly number of PCOS-related medical visits to be 493 per 100,000 women ages 10 to 60 years. Jason found that patients seen for PCOS-related visits tended to be younger, obese, weigh more, and more likely to be ordered or provided with diet and nutrition counseling, compared to patients seen for non-PCOS-related visits. The findings are consistent with the recommendations from the Androgen Excess and Polycystic Ovary Syndrome Society and Endocrine Society that lifestyle management (i.e., diet and exercise) should be the first line of treatment for obese women with PCOS.^20^^,^ 21 However, further research is needed to understand additional patient characteristics and behavioral health services associated with PCOS-related medical visits. In addition, Jason was unable to calculate national estimates of PCOS-related visits in which the patient also had a diagnosis of diabetes because these data did not meet the National Center for Health Statistics criteria necessary to provide reliable estimates. This underscores the need to further examine whether NAMCS and NHAMCS are suitable to provide reliable estimates of patient characteristics and behavioral health services associated with PCOS-related medical visits.

### Behavioral health interventions

The U.S. Preventive Services Task Force recommends screening for depression among women, people with chronic illness, and those with additional mental health disorders.^22^ Furthermore, the Centers for Disease Control and Prevention recommend integrating mental health promotion and mental illness prevention with chronic disease prevention.^23^ The comorbidity (co-occurrence) of chronic mental and physical illness poses significant burdens to the US population^24^ and the health care system.^25^ This is because comorbidity tends to complicate treatment, affect prognosis, and limit quality of life.^26^ For instance, depression, one of the most common mental illnesses seen in primary care,^27^ is associated with poor adherence to treatment, increased symptoms, medical costs, and impairment of daily life (e.g., walking, ability to work), among patients with chronic illness.^28^ About one fifth (22.4%) of adults in mental health care drop out of treatment, and patients with psychiatric comorbidity are likely to drop out by the third visit.^29^

Research on PCOS suggests that PCOS is comorbid with depression. For instance, research with women in the USA (*M* = 35.2 years old) recruited from reproductive endocrine departments and private practices in the Pittsburgh, Pennsylvania area estimated the prevalence of clinical depression, based on women’s self-report on the Beck Depression Inventory, to be about 30% among women with PCOS, compared to 17% among controls.^30^ Among women with PCOS, those with higher body mass index, more children, and less education were at greatest risk for mild to moderate depressive symptoms. In addition, low-income patients with a diagnosis of PCOS based on the State of Louisiana Medicaid claims were more likely to have a diagnosis of anxiety and depression compared to a control group.^31^ Research with trained clinicians has also found that women with PCOS in Sweden tend to have a higher lifetime incidence of depressive episodes and social phobia and are more likely to attempt suicide compared to age-matched controls.^32^ Furthermore, Skype interviews with women with PCOS living in Australia indicate that managing PCOS in addition to other health conditions can be particularly challenging, leading some women to develop depressive symptoms, self-harm, and suicidal ideation.^33^

The Australian Evidence-based Guideline for the Assessment and Management of PCOS recommends screening and treating patients for anxiety, depression, eating disorders, and poor body image associated with PCOS symptoms such as hirsutism, acne, obesity, and impaired fertility. Depression screening, for instance, can prompt intervention to improve emotional well-being, motivation, and self-efficacy necessary to make lifestyle changes and manage PCOS.^34^

### Health education

Health education is necessary to help patients with PCOS understand their condition and manage their symptoms.^35^ Women diagnosed with PCOS report seeing multiple health care providers before receiving a diagnosis, feeling dissatisfied with the information provided to them at the time of diagnosis,^36^ and look to the Internet for information on PCOS.^37^ In addition, patients’ perceptions of the quality of health-related information they receive about PCOS are associated with their quality of life.^38^ Research with adolescents with PCOS in the USA found that 32% of adolescents did not understand PCOS, 48% understood somewhat, and 20% understood the condition well.^39^ Additional research with adolescents with PCOS has found that adolescents express “worry,” “shock,” “disbelief,” and “panic” at the time of diagnosis; report receiving inadequate information from providers, particularly in primary care; and do not understand complex terminology. 40 ^(p. 579)^ Health education, combined with individualized treatment plans, influences patient motivation to implement lifestyle changes to prevent health risks associated with PCOS.^41^ Health education in the areas of family planning and contraception, growth and development, stress management, and tobacco use is relevant to the care of patients with PCOS, particularly as women have expressed strong interest in nonmedication treatment for PCOS.^42^

#### Family planning and contraception

Health education in the areas of family planning and contraception is critical for patients with PCOS as the condition affects the reproductive system. Examples of the need for health education in these areas include women’s lack of awareness of infertility and its relationship to PCOS,^43^ adolescents’ confusion about PCOS-related infertility and worries about their ability to have children in the future,^39^ effects of contraceptives on PCOS symptoms,^44^ women’s concerns that contraceptives to induce menstruation result in “artificial femininity,” 45 ^(p. 356)^ and the role of oral contraceptives in reducing the risk of developing PCOS-related endometrial cancer.^46^

#### Growth and development

Research using 1993–2002 NAMCS and NHAMCS has found that increased awareness of childhood obesity as a public health problem is associated with a shift in the provision of general growth/development counseling to obesity-related counseling.^47^ Similarly, PCOS-related growth and development education can help patients understand the consequences of PCOS across the lifespan^12^^,^ 13 ^,^ 48 and learn how to manage their symptoms to prevent risks associated with PCOS such as type 2 diabetes and impaired fertility. When working with adolescents with PCOS, providers should keep in mind that adolescents appreciate when providers direct discussion toward them and not just their parents.^40^ Although PCOS is described as one of the most common endocrine disorders in women of reproductive age,^2^ health risks associated with PCOS can persist postmenopause. For instance, women with PCOS ages 61 to 79 years tend to have a higher prevalence of hypertension and triglycerides,^49^ compared to women without PCOS. In addition, women with PCOS ages 43 to 59 years have a greater likelihood of insulin resistance,^50^ compared to similarly aged women without PCOS. These risk factors, combined with cigarette smoking, obesity, and type 2 diabetes, make women with PCOS susceptible to cardiovascular disease.^20^

#### Stress management

Stress management is an important component of health promotion.^51^ Chronic stress influences how individuals cope with and manage poor health,^52^ and women with PCOS may be more reactive to stressful experiences (e.g., higher cortisol levels and heart rate) compared to women without PCOS matched by body mass index.^53^ Increased awareness of the associations between stress, coping, mental health, and quality of life among patients with PCOS can prompt additional mental health screening and stress management counseling. For instance, research with 2005–2010 NAMCS and NHAMCS found that depression screening among adolescents was more likely to occur if stress management or other mental health counseling was provided.^54^ Among women with PCOS, poor coping skills are associated with greater levels of anxiety, depression, and lower quality of life.^55^ Patients with PCOS report that stress management techniques in exercise programs increase sense of control over one’s body and life.^56^ Incorporating coping strategies into health education and psychosocial support groups^55^ and the use of nurse-led peer support groups^57^ are promising approaches for improving health education and self-management among patients with PCOS. In addition, there is increased interest in yoga programs that incorporate stress management techniques to reduce anxiety and improve quality of life among adolescents with PCOS^58^^–^ 60 and mindfulness approaches that reduce stress and symptoms of depression and anxiety and increase quality of life among women with PCOS.^61^

#### Tobacco use

The Endocrine Society recommends that adolescents and women with PCOS be screened for cardiovascular disease risk factors, including cigarette smoking.^21^ Women with PCOS who smoke tend to have higher triglyceride levels, compared to nonsmokers, even after adjusting for body mass index.^62^ In addition, total cholesterol is positively associated with higher amount and duration of smoking among women with PCOS.^63^ Furthermore, among obese women with PCOS who received infertility treatment, couples in which both partners smoked had a lower likelihood of live birth, compared to nonsmokers.^64^ Patients with PCOS can benefit from health education and counseling on the associations between increased age, smoking, metabolic syndrome, and cardiovascular disease.

This study examines the suitability of NAMCS and NHAMCS to produce reliable national estimates of patient demographics and behavioral health care associated with PCOS-related medical visits. Specifically, this study examines items on mental health and health education. Mental health care items include whether the patient had a diagnosis of depression at the time of the visit, a depression screening exam was ordered or provided, a mental health provider was seen, psychotherapy was ordered or provided, and other mental health counseling was ordered or provided at the time of the visit. Items on health education ordered or provided are in the areas of family planning/contraception, growth/development, stress management, and tobacco use/exposure.

The findings can identify suppressed data, which refers to data not reported due to their inability to meet criteria to provide reliable estimates.^65^ Reports that evaluate the country’s progress in addressing health and health care disparities, such as the National Healthcare Disparities Report and Healthy People, suppress data that do not meet reliability criteria.^65^^–^ 67 Suppressed data can limit our understanding of patient characteristics and behavioral health care associated with PCOS-related visits.

## Method

### National health care surveys

This study uses annual, cross-sectional data from the 2005–2010 NAMCS and NHAMCS. NAMCS and NHAMCS are part of the National Health Care Surveys conducted by the National Center for Health Statistics, Centers for Disease Control and Prevention.^68^ NAMCS and NHAMCS measure several aspects of health care provision and utilization including patient sociodemographic characteristics, diagnoses, providers seen, and services ordered or provided.

Medical visit information is collected from health care providers in hospitals and clinics located in the 50 states and the District of Columbia.^69^^,^ 70 Together, NAMCS and NHAMCS provide a nationally representative sample of ambulatory medical visits in the USA.^69^ Ambulatory or outpatient care refers to care in which an individual seeking health services is not currently admitted (hospitalized) to any health care institution on the premises.

### NAMCS

NAMCS collects data on ambulatory care provided by nonfederally employed, office-based physicians primarily engaged in direct patient care. Examples of these office settings include private, solo, or group practices; community health centers; mental health centers; and family planning clinics. NAMCS utilizes a three-stage design that involves probability samples of primary sampling units (PSUs) (e.g., a county, a group of counties), physician practices within PSUs, and medical visits within physician practices.^69^ There were a total of 180,086 patient record forms in the 2005–2010 NAMCS.

### NHAMCS

NHAMCS collects data on ambulatory care in emergency and outpatient departments (EDs and OPDs) of noninstitutional, general short-stay (less than 30 days) hospitals, excluding Federal, military, and Veterans Administration hospitals. Outpatient department refers to a hospital facility where nonurgent ambulatory medical care is provided under the supervision of a physician. Emergency department refers to a hospital facility that provides unscheduled outpatient services to patients whose conditions require immediate care and is staffed 24 hours a day. Emergency departments open less than 24 hours a day are included as part of the hospital’s outpatient department. An emergency service area is an area within the emergency department where emergency services are provided.^70^

NHAMCS utilizes a four-stage design that involves probability samples of PSUs, hospitals within PSUs, clinics/emergency service areas within outpatient/emergency departments, and patient visits within clinics/emergency service areas.^70^ From 2005 to 2010, there were a total of 208,956 NHAMCS patient record forms from emergency departments and 201,730 patient record forms from outpatient departments.

NAMCS and NHAMCS also contain income and education data associated with patients’ residential zip code based on 2000 Census data. In the public use files, these data are categorized in quartiles (e.g., median household income) or grouped percentages (e.g., percent of population below the poverty level).^71^

Personally identifying information, such as patient names, street addresses, and social security numbers are not collected in NAMCS and NHAMCS to ensure confidentiality.^69^^,^ 70 This study used publicly available, deidentified data and was deemed exempt from review by the University of Michigan Institutional Review Board.

## Measures

This study used data starting from 2005 because that is the year mental health items such as depression at the time of the visit and depression screening ordered or provided were first introduced in NAMCS and NHAMCS OPD. The item on mental health provider seen was first introduced in the 2007 NAMCS and NHAMCS OPD and in the 2009 NHAMCS ED.^72^^–^ 74

### Patient characteristics

Patient sex and race were based on information on the medical record or observation or knowledge of the patient. Patient sex and race imputed by the National Center for Health Statistics staff in the 2005–2010 NAMCS and NHAMCS were used in this study.^69^^,^ 70

#### Sex

Sex was coded 1 = female and 0 = male.

#### Race

Race was coded 1 = White (a person having origins in any of the original peoples of Europe, the Middle East, or North Africa) and 2 = Black or Other (Black refers to a person having origins in any of the black racial groups of Africa. Other referred to Asian, Native Hawaiian/Other Pacific Islander, and American Indian/Alaska Native). Although NAMCS and NHAMCS contained imputed ethnicity variables on ethnicity (Hispanic, not Hispanic), these variables did not have enough PCOS-related visits to produce reliable estimates.

#### Age

Patient age in years was derived from patient date of birth.

#### Expected source of payment

Private insurance as the expected source of payment was coded as 1 = Yes and 0 = No. The variable that contained a hierarchy of payment categories (private insurance, Medicare, Medicaid or Medicaid/SCHIP, worker’s compensation, self-pay, no charge/charity, other, and unknown) was not used because it did not meet reliability criteria.

### Visit characteristics

#### Physician’s diagnosis

Diagnoses were coded according to the International Classification of Diseases, 9th Revision, Clinical Modification (ICD-9-CM).^75^ Up to three diagnoses were collected from the provider. In the present study, medical visits were considered PCOS-related if they had a medical code of 256.4 on any of the three provider diagnoses.

#### Depression

In addition to the diagnoses described above, the provider also indicated the presence or absence of other conditions at the time of the visit. One of these conditions was depression. Depression was defined in NAMCS and NHAMCS OPD documentation as affective disorders and major depressive disorders, such as episodes of depression reaction, psychogenic depression, and reactive depression. Absence of depression at the time of the visit was coded 0 = No and presence of depression 1 = Yes.^76^

#### Depression screening exam

This item indicated whether a depression screening was ordered or provided at the time of the visit and was coded as 0 = No and 1 = Yes.

#### Psychotherapy ordered or provided

Psychotherapy refers to all treatments involving the intentional use of verbal techniques to explore or alter the patient’s emotional life in order to effect symptom reduction or behavior change. Psychotherapy ordered or provided was coded 0 = No and 1 = Yes.

#### Other mental health counseling

Other mental health counseling refers to general advice and counseling about mental health issues and education about mental disorders. This includes referrals to other mental health professionals for mental health counseling.^69^^,^ 70 Other mental health counseling was coded 0 = No and 1 = Yes.

#### Mental health provider seen

Mental health provider refers to psychologists, counselors, social workers, and therapists who provide mental health counseling, excluding psychiatrists.^72^^,^ 74 Mental health provider seen was coded 0 = No and 1 = Yes.

#### Health education ordered or provided

The following types of health education ordered or provided at the visit were included in this study. Each type of health education ordered or provided was coded 0 = No and 1 = Yes.

- **Family planning/contraception**. Information given to the patient to assist in the conception or intended to help the patient understand how to prevent conception.
- **Growth/development**. Topics related to human growth and development.
- **Stress management**. Information intended to help patients reduce stress through activities such as exercise, biofeedback, and yoga. It also includes referrals to other health professionals for the purpose of coping with stress.
- **Tobacco use/exposure**. Information given to the patient on issues related to tobacco use in any use/exposure form, including cigarettes, cigars, snuff, and chewing tobacco, and on the exposure to tobacco in the form of “secondhand smoke.” This also includes information on smoking cessation and prevention of tobacco use as well as referrals to other health professionals for smoking cessation programs.

### Sociogeographic characteristics

Starting in 2006, patient zip code in NAMCS and NHAMCS was used with the data from the 2000 Census to determine education, income, and poverty measures.^77^^,^ 78 In the public use files, income and education data were categorized in quartiles. These variables were collapsed as follows in order to meet reliability criteria:

- *Percent poverty in patient’s zip code* was coded 1 = less than 10% and 2 = greater than or equal to 10%.
- *Median household income in patient’s zip code* was coded 1 = less than or equal to $40,626 and 2 = greater than or equal to $40,627.
- *Percent population with bachelor’s degree or higher in patient’s zip code* was coded 1 = less than or equal to 19.66% and 2 = greater than or equal to 19.67%.

## Analyses

The 2005 to 2010 NAMCS and NHAMCS ED and OPD data were combined to produce national estimates of PCOS-related ambulatory medical care in the USA. Analyses were conducted using Stata 13.1 using complex survey commands.^79^ Patient sampling weights were used to obtain national estimates of patient visits from 2005 to 2010. The estimated average annual number of patient visits was obtained by dividing the weighted total estimate of patient visits by 6, the number of years of survey data used. The “svyset” command in Stata was used to define the complex sample design and sampling weights. Between 2005 and 2010, all PCOS-related visits were made by 11- to 60-year-old females. To conduct analyses on this subpopulation, dummy variables indicating the desired subpopulation (11- to 60-year-old females) were created, and the “subpop()” function was used to identify the desired subpopulation. This option takes into account the entire data set in Stata, identifies the full complex sample design, and performs analyses on the desired subpopulation of data. The “svy: mean” command was used to compute the estimated mean patient ages and standard errors for PCOS and non-PCOS-related visits. The “lincom” command was used after this to test for a difference in mean age for these two subgroups while still accounting for the complex design and sampling. The “svy: tabulate” command was used to derive the percentage distributions of PCOS and non-PCOS-related visits by patient and visit characteristics as well as the associated standard errors. The Rao-Scott chi-square test was used to test for relations between visit type (PCOS and non-PCOS-related visits) and patient age, race, private insurance, and geographic characteristics, to account for the complex sampling design. An alpha level of 0.05 was the criterion for statistical significance.

According to the National Center for Health Statistics, reliable estimates are based on at least 30 unweighted records and a relative standard error (standard error divided by estimate) less than 30%. Both conditions should be met before estimates are considered reliable.^80^ Relative standard error is calculated by dividing the standard error of the estimate by the estimate itself, then multiplying that result by 100 to obtain a percent. Relative standard error is expressed as a percent of the estimate. For example, if the estimate of White patients seen for PCOS-related visits is 70% and the standard error of the estimate is 6%, the relative standard error of the estimate = (6/70) * 100, or 8.6%. Due to the limited sample size for PCOS-related visits, further advanced multivariate analyses were not conducted as this would have yielded unreliable estimates.

## Results

Between 2005 and 2010, there were 246 unweighted medical records (out of 590,772 total unweighted records) that had a diagnostic code of PCOS. These 246 visits represent 3,526,716 weighted PCOS-related visits. The mean yearly rate of PCOS-related visits was 587,786 visits; that is, 306 per 100,000 women ages 11 to 60 years old.

Table 1 describes patient characteristics that met reliability criteria. PCOS-related visits were made largely by patients who were younger, on average 10 years younger, than patients seen for non-PCOS-related visits. A larger proportion of White patients (86.9%), who paid with private insurance (82.9%), were seen for PCOS-related visits. In addition, a greater proportion of PCOS-related visits were made by patients living in zip codes with percent poverty level greater than 10%. There were no statistically significant associations between visit type and median household income in patient’s zip code or percent population with a bachelor’s degree or higher in the patient’s zip code.

**Table 1:** Patient sociodemographic and sociogeographic characteristics: comparisons between PCOS- and non-PCOS-related visits in the USA among females ages 11 to 60 years, 2005–2010

	PCOS-related visits				Non-PCOS-related visits				
	Frequency of unweighted records	Weighted average visits per year	% visits per year	Relative standard error (%)	Frequency of unweighted records	Weighted average visits per year	% visits per year	Relative standard error (%)	*p* value
Patient sociodemographic characteristics									
Age (years)	(Mean = 28.3, SD = 7.0)				(Mean = 38.2, SD = 14.4)				*p < 0.001*
Race									*p < 0.10*
White	197	510,996	86.9	3.8	438,002	333,750,364	80.1	1.2	
Black or Other	49	76,790	13.1	25.4	149,349	83,148,747	19.9	4.9	
Payment type									
Private insurance									*p < 0.001*
Yes	158	487,224	82.9	4.9	261,794	268,279,260	64.4	1.0	
No	88	100,562	17.1	23.6	325,557	148,619,851	35.6	1.8	
Geographic characteristics									
Percent poverty in patient’s zip code									*p = 0.0450*
<10%	97	157,294	38.1	18.5	208,370	170,831,626	52.0	3.0	
≥10%	103	255,238	61.9	11.4	263,506	157,411,429	48.0	3.2	
Median household income in patient’s zip code									*p* = 0.5708
≤$40,626	96	179,387	43.5	14.7	254,822	154,079,470	46.9	3.2	
≥$40,627	104	233,146	56.5	11.3	217,121	174,217,619	53.1	2.9	
Percent population with bachelor’s degree or higher in patient’s zip code									*p* = 0.5596
≤19.66%	94	184,358	44.7	15.4	250,784	160,201,707	48.8	2.6	
≥19.67%	106	228,175	55.3	12.4	220,926	168,052,693	51.2	25	

Statistically significant *p* values based on *t* test or *χ* ^2^ test in italics

Table 2 describes the reliability criteria for items on patient characteristics, and Table 3 describes the reliability criteria for items on mental health services and health education ordered or provided.

**Table 2:** Description of reliability criteria for patient characteristics among females ages 11 to 60 years seen for PCOS-related visits, 2005–2010 NAMCS and NHAMCS

	PCOS-related visits	
	Frequency of unweighted records	Relative standard error (%)
Patient sociodemographic characteristics		
Race/ethnicity		
Non-Hispanic White	117	8.3
Non-Hispanic Black	*16*	*43.1*
Hispanic	*22*	*29.1*
Asian	*5*	*54.6*
Native Hawaiian/Other Pacific Islander	*4*	*91.4*
American Indian/Alaska Native	*1*	*101.5*
Multiple races	*3*	*72.4*
Payment type		
Private insurance	158	4.8
Medicare	*0*	*0*
Medicaid/SCHIP	*49*	*35.5*
Worker’s compensation	*0*	*0*
Self-pay	*19*	*49.1*
No charge	*5*	*65.4*
Other	*7*	*59.5*
Unknown	*4*	*89.9*
Patient sociogeographic characteristics		
Percent poverty in patient’s zip code		
Quartile 1 (less than 5%)	*39*	*35.4*
Quartile 2 (5 to 9.99%)	58	21.8
Quartile 3 (10 to 19.99%)	72	15.4
Quartile 4 (20% or more)	*31*	*47.1*
Median household income in patient’s zip code		
Quartile 1 (below $32,793)	*42*	*31.5*
Quartile 2 ($32,794–$40,626)	54	20.2
Quartile 3 ($40,627–$52,387)	56	17.4
Quartile 4 ($52,388 or more)	48	24.7
Percent population with bachelor’s degree or higher in patient’s zip code		
Quartile 1 (less than 12.84%)	*45*	*34.9*
Quartile 2 (12.84 to 19.66%)	49	23.7
Quartile 3 (19.67 to 31.68%)	61	18.2
Quartile 4 (31.69% or more)	45	24.7

Items in italics do not meet the National Center for Health Statistics reliability criteria. Estimates should be based on at least 30 unweighted records and estimates with a relative standard error (standard error divided by estimate) less than 30%. Both conditions should be met before estimates are considered reliable

**Table 3:** Description of reliability criteria for mental health care services and health education ordered or provided among females ages 11 to 60 years seen for PCOS-related visits, 2005–2010 NAMCS and NHAMCS

	PCOS-related visits	
	Frequency of unweighted records	Relative standard error (%)
Mental health care services		
Depression screening ordered or provided		
Yes	*6*	*59.8*
No	231	2.1
Depression diagnosis at the time of the visit		
Yes	*23*	*35.2*
No	214	5.8
Psychotherapy ordered or provided		
Yes	*4*	*72.1*
No	233	2.4
Mental health provider seen		
Yes	*1*	*97.9*
No	155	0.2
Other mental health counseling ordered or provided		
Yes	*2*	*82.6*
No	235	0.6
Health education ordered or provided		
Family planning/contraception		
Yes	*7*	*47.1*
No	68	7.7
Growth/development		
Yes	*4*	*65.2*
No	233	3.7
Stress management		
Yes	*8*	*70.7*
No	229	0.5
Tobacco use/exposure		
Yes	*11*	*43.9*
No	226	2.1

Items in italics do not meet the National Center for Health Statistics reliability criteria. Estimates should be based on at least 30 unweighted records and estimates with a relative standard error (standard error divided by estimate) less than 30%. Both conditions should be met before estimates are considered reliable

## Discussion

This study is the first to examine patient sociodemographics and mental health services and health education ordered or provided at PCOS-related visits using 2005–2010 NAMCS and NHAMCS. The number of unweighted records with a diagnosis of PCOS in this study (*n* = 246) is within the range of the number of PCOS-related records Jason found (*n* = 175) using the 2003–2008 NAMCS and NHAMCS data.^19^

Overall, the results of the current study suggest that larger sample sizes of PCOS-related visits are necessary to understand potential disparities in PCOS-related visits, particularly by insurance coverage and race and ethnicity, and to obtain reliable estimates of mental health and health education services.

### Patient sociodemographics

#### Age

The ages of patients seen for PCOS-related visits (11–60-year olds) in this study reflect the health needs of patients with PCOS across the life span. The finding that patients seen for PCOS-related visits were significantly younger than patients seen for non-PCOS-related visits is consistent with Jason’s findings.^19^ The results suggest that health services to manage PCOS and prevent its complications in later life are important, not only among women of reproductive age but also among children, adolescents, and women postmenopause.

#### Insurance type

The finding that the proportion of PCOS-related visits paid with private insurance was higher than that of PCOS-related visits paid without private insurance mirrors national trends associated with insurance coverage and health care access and utilization. For instance, according to the National Healthcare Disparities Report, people with private insurance are about twice as likely to have a specific source of ongoing care compared to the uninsured.^67^ In addition, previous research with NAMCS and NHAMCS has found delayed transitions in care among young adults with chronic disease and with public or no health insurance.^81^ Continuous access to quality, affordable health services is especially important for patients with a chronic condition such as PCOS that requires long-term monitoring and care.

#### Race

A large proportion of PCOS-related visits were by White women. This is concerning as racial and ethnic minority women may be more susceptible to PCOS and its complications, compared to White women. For instance, Black women with PCOS tend to have high rates of obesity and hypertension, followed by Hispanic women; yet, screening for metabolic disorders (e.g., obesity, high cholesterol, blood pressure, and type 2 diabetes) among racial and ethnic minority women, and women with PCOS in general, tends to be low.^82^

### Behavioral health services

Reliable estimates of PCOS-related mental health and health education services are necessary to examine potential disparities in PCOS-related care. Existing health care disparities shed some light on why some groups have a higher likelihood of referral to, or receipt of, mental health and health education/counseling services.

#### Mental health

In the USA, mental health problems are less likely to be recognized and managed in primary care, compared to specialist care, due to providers’ lack of time, skills, or knowledge of mental health problems.^25^ In addition, research using NAMCS and NHAMCS has found that Hispanic adolescents are less likely to be screened for depression than non-Hispanic, White adolescents,^54^ and disparities in diagnosis and treatment of anxiety and depression among African American and Hispanic adult patients in primary care visits persist.^83^ Furthermore, research conducted by the Substance Abuse and Mental Health Services Administration, Health Resources and Services Administration, and Centers for Medicare and Medicaid Services has identified lack of reimbursement for mental health services as one of several barriers to the provision of mental health services in primary care settings.^84^

#### Health education/counseling

Research with the 2005–2007 NAMCS data has also found that while obese patients may receive diet, exercise, and weight reduction education, they are less likely to receive tobacco education and psychotherapy referral compared to normal weight patients, suggesting that behavioral health services may be overlooked if weight alone is seen as the priority.^85^ Research with 2005–2010 NAMCS and NHAMCS also suggests that overweight/obese pregnant women are less likely to receive diet/exercise counseling compared to overweight/obese, nonpregnant women.^86^ In addition, disparities in reproductive health services persist, particularly among young, immigrant women with low education level and underinsured.^87^ Furthermore, disparities in access to effective treatment for infertility exist partly due to lack of research on biological causes of infertility, including PCOS, by race and ethnicity; prolonged duration of infertility prior to seeking medical advice; and economic barriers such as inability to pay for fertility treatment.^88^ Research using 2009–2010 NAMCS has also found low rates of family planning during visits made by reproductive-aged women, including women with risk factors for cardiovascular disease.^89^

This study had some limitations. First, as Jason noted, the diagnostic code for PCOS may not have captured unrecognized cases of PCOS.^19^ For instance, the lack of standardized, universal criteria for diagnosing PCOS can lead to inconsistencies and delays in diagnosis. According to the 2012 NIH Evidence-based Methodology Workshop on PCOS, “The use of multiple classification systems is confusing and delays progress in understanding the syndrome. It also hinders the ability of clinicians to partner with women to address and manage the health issues that concern them.” 2 ^(p. 2)^. Second, health education and mental health services ordered may not have been provided at the time of the visit. However, these items capture an intent to provide these services.^90^ In addition, the cross-sectional nature of the surveys used eliminated the ability to assess whether patients had previously been referred for health education and mental health services or whether they had in the past discussed preference for these services.

## Implications for Behavioral Health

In the USA, low-income populations, racial and ethnic minorities, women, and those with chronic disease are considered priority populations. The Agency for Healthcare Research and Quality defines priority populations as groups with health care needs requiring special attention to reduce disparities in health care among these populations.^67^ Health care disparities refer to differences in health coverage, access to care, and quality of care between populations.^91^ Low-income, racial and ethnic minority adolescents and women with PCOS should be included in this priority.

The findings underscore the importance of strengthening national surveillance to further understand patient demographic characteristics and behavioral health care associated with PCOS-related visits. Effective data collection is an important part of ensuring that people’s health and mental health needs are met.^25^ Effective surveillance systems should have sufficiently large sample sizes, even after combining multiple years of data, to make national estimates and conduct analyses of subgroups.^92^ In this study, items on mental health services and health education ordered or provided did not meet reliability criteria, even after combining 6 years of data. Indeed, reviews of existing data sources find that larger national sample sizes are necessary to identify health disparities for major racial, ethnic, and socioeconomic subgroups.^93^ As researchers involved in developing the first National Healthcare Disparities Report stated, “Disparities in data available to assess the health care of different racial, ethnic, and socioeconomic groups compound the problem of disparities in access to and quality of care.” 65 ^(pI-15)^.

Increased awareness of PCOS and the needs of patients with PCOS can lead to inclusion of PCOS-related items in national health surveys and larger sample sizes of PCOS-related visits in the National Health Care Surveys. For instance, as a result of a partnership between the National Center for Health Statistics and the Health Resources and Services Administration, NAMCS oversampled community health centers for the first time in 2006.^94^ The oversampling increased the reliability of estimates for visits to community health centers and led to important findings regarding the demographic characteristics of patients visiting community health centers.^94^^–^ 96 Efforts to collect PCOS data on a large scale is also likely to require the support and endorsement of groups knowledgeable about PCOS research and practice. For instance, in 2010, NAMCS began collecting data on blood glucose and other laboratory tests related to cardiovascular disease after the American Heart Association released a scientific statement recommending these data be collected to track progress of heart disease and stroke prevention and management. Including these items in NAMCS provided “a low-cost approach to enhance national surveillance for cardiovascular disease”. 69 ^(p. 116)^ Note that a new item may not be added to the health care surveys without a sponsoring agency to fund the additional items or if no expert meeting recommends the addition of items. In addition, the use of paper surveys means that an item will likely be omitted if a new item is to be added because the paper format limits the total number of items that can be collected.^97^ Developing surveillance systems to understand patient demographics and behavioral health services for patients with PCOS is a major step in addressing this public health problem for women.
